# Family physicians’ attitude and practice of infertility management at primary care - Suez Canal University, Egypt

**DOI:** 10.11604/pamj.2013.15.106.1762

**Published:** 2013-07-23

**Authors:** Hebatallah Nour Eldein

**Affiliations:** 1 Department of Family Medicine, Faculty of Medicine, Suez Canal University, Egypt

**Keywords:** Family physician, attitude, practice, infertility management, primary care

## Abstract

**Introduction:**

The very particular natures of infertility problem and infertility care make them different from other medical problems and services in developing countries. Even after the referral to specialists, the family physicians are expected to provide continuous support for these couples. This place the primary care service at the heart of all issues related to infertility. The aim of the work: to improve family physicians' attitude and practice about the approach to infertility management within primary care setting.

**Methods:**

This study was conducted in the between June and December 2010. The study sample comprised 100 family physician trainees in the family medicine department and working in family practice centers or primary care units. They were asked to fill a questionnaire about their personal characteristics, attitude, and practice towards support, investigations, and treatment of infertile couples.

**Results:**

Hundred family physicians were included in the study. They were previously received training in infertility management. Favorable attitude scores were detected among (68%) of physicians and primary care was considered a suitable place for infertility management among (77%) of participants. There was statistically significant difference regarding each of age groups, gender and years of experience with the physicians′ attitude. There was statistically significant difference regarding gender, perceiving PHC as an appropriate place to manage infertility and attitude towards processes of infertility management with the physicians′ practice.

**Conclusion:**

Favorable attitude and practice were determined among the study sample. Supporting the structure of primary care and evidence-based training regarding infertility management are required to improve family physicians' attitude and practice towards infertility management.

## Introduction

Infertility can be defined as the failure to achieve a pregnancy within one year of regular unprotected intercourse [1, 2]. The very particular nature of infertility problem and of infertility care makes them different from other medical problems and services in developing countries. On the other hand, negative psychosocial, sociocultural, and economical consequences of childlessness are often more pronounced compared with Western societies [3, 4].

Infertility is a problem of global proportions; the exact prevalence of infertility in developing countries is unknown due to a lack of registration and well-performed studies [5]. It is affecting on average 8-12 percent of couples worldwide. In some societies, however-particularly those in the &infertility belt&34; of sub-Saharan Africa as many as one-third of all couples are unable to conceive [6]. Prevalence of infertility is 10.4 % among women between 15-49 years in rural areas of Kafr ElSheikh - Egypt [7]. There is a wider prevalence range in resource-poor countries, possibly due to different country-specific factors, such as the prevalence of sexually transmitted diseases, age at delivery, and political factors [3].

In Germany, Many family physicians regard fertility counseling out of their scope of practice, although key elements in the care of involuntarily childless couples fall within the theoretical framework of family practice [8]. In United Kingdom, each general practitioner sees approximately two infertile couples each year. Despite the relative lack of opportunity to rehearse the skills of new technologies necessary to manage infertile couples, a basic understanding will assist in the general practitioners advocacy role [9]. The primary care physician who provides preventive care can initiate the diagnostic evaluation and can treat some causes of infertility [10]. The initial investigations commonly performed by general practitioners include semen analysis, serum mid-luteal progesterone and serum follicle stimulating hormone. Management strategies for the general practitioners include optimising general health, weight loss for obese infertile women, ovulation induction with clomifene and expectant management for young women with no identified cause for their delayed fertility [9].

The treating physician who is counseling the couple regarding their infertility must be familiar with the causes, investigations and the treatment options available. Even after the referral to specialists, the general practitioners are expected to provide continuous support for these couples [11]. This place the primary care service at the heart of all issues related to infertility [12].

The quality of infertility care is dependent upon adequate material resources and the appropriate use of it [13]. By following evidence-based, management protocol, infertile couples will have a good chance to start up their treatment in the proper way at early time with enough financial support through reducing money spent on unnecessary investigations [10]. The aim this study was to assess family physicians′ attitude and practice of infertility management to improve the care of infertile couple within primary care settings.

## Methods

**Study design:** This was a cross sectional-descriptive study.

### Sampling and subjects

The study was carried in Family Medicine department-Suez Canal University between June and December 2010. It included all family physician trainees (100 physicians) working in family medicine centers and primary care units. The participant included 53 (assistant lecturers, residents, diploma) physicians and 47 Egyptian fellowship physicians. A validated modified questionnaire was self administered by the family physicians during the break of the scientific day in the department and the researcher was on hand to explain any incomprehensible question.

The questionnaire was divided into six sections:

- Section 1: Personal data of family physicians and wether they practiced infertility management or not
- Section 2: Physicians′ attitude regarding the appropriateness of Primary Health Care (PHC) as a place for infertility management and the difficulties of providing this service
- Section 3: Physicians′ attitude and practice concerning supportive treatment and proposals for infertile couples
- Section 4: Physicians′ attitude and practice concerning request for laboratory investigations
- Section 5: Physicians′ attitude and practice about treatment of infertillity cases. Attitude scores of processes of infertility management within PHC were assessed in sections 3, 4, & 5 by giving 1 point to each agree response and zero to not agree. Total score was 31 points. Favorable attitude was considered for scores that were equal or above the mean. Practice of each process was assessed also in sections 3, 4 and 5 as practiced or not
- Section 6: Physicians′ suggestions about improving the service of infertility management in primary care were gathered from practicing physicians

The questionnaire was developed depending on the designed questionnaire by Hassa et al (2005) [14] and modified by the researcher according to the National Institute of Clinical Excellence NICE guideline - Fertility (2004) [15]. Modifications were applied to physicians′ opinions by substituting the first item (about rubella vaccination as it was not included in the Egyptian premarital examination program) by meeting the couple. History and physical examination were added as the second item. The verbs in items (11-13) about ultrasonography (U/S) were modified to request instead of perform as U/S was not available in some of the family practice centers at the time of the study. Item 14 (to measure basal body temperature ) was excluded as it is not recommended in recent guidelines.

A pilot study was conducted to 10 % of the study population (were not included in the final results) to test the relevancy of the questionnaire to the aim of the work, determine if the questions asked were understood by the respondents or not, perform any modification needed and determine the time required for the questionnaire. The necessary modifications were done, so some statements were reworded. The average interviewing time was 15 minutes. Validity and reliability of the questionnaire were tested.

**Data analysis:** Data were analyzed using Software Package for Social Sciences (SPSS) version 18. Data were presented using descriptive statistics in the form of frequencies and percentages for qualitative variables. Chi-square or Fisher's exact test was used to test the relationship between categorical variables and the level of significance was considered statistically significant if p-value is (< 0.05). Data were presented in suitable tables and figures.

**Ethical considerations:** The study was approved by the ethics committee of the faculty of medicine, Suez Canal University and has been performed in accordance with the ethical standards laid down in the 1964 Declaration of Helsinki. All physicians who accept the participation were signed informed consent prior to their inclusion into the study. The questionnaire was anonymous; no critical questions and confidentiality of data were preserved.

## Results

Out of 100 family physicians, (61%) of them were in the age group of (24-29). Female physicians were more than two thirds (71%). Most of the physicians were not qualified by Master or Diploma degree (74%). About two third of family physicians were with less than 5 years of experience (62%) and two thirds of them were working in family practice centers of Suez Canal University (66%) and in rural community (66%). 100% of the participants had no other specialty than family medicine.

Table 1 shows the relationship between personal characteristics of family physicians and their attitude towards processes of infertility management in primary care. Out of 100 studied physicians, 68 ones had favorable attitude. There were statistically significant difference regarding each of age groups, gender, and years of experience with the physicians′ attitude (p < 0.05).

**Table 1:** The relationship between personal characteristics of family physicians and their attitude towards processes infertility management in primary care

		Attitude towards infertility management						x^2^	P value
		Favorable		Unfavorable		Total			
		N=68	%	N=32	%	N=100	%		
Age groups	24-29	35	57.4%	14	42.6%	61	61.0%	8.167 Fisher Exact	0.017*
	30-35	24	85.7%	7	14.3%	28	28.0%		
	>35	9	81.8%	4	18.2%	11	11.0%		
Gender	Male	13	44.8%	16	55.2%	29	29.0%	8.964	0.003*
	Female	55	77.5%	16	22.5%	71	71.0%		
Qualification	Master degree	21	80.8%	5	19.2%	26	26.0%	2.633	0.105
	Others	47	63.5%	27	36.5%	74	74.0%		
Place of work	FPC	46	69.7%	20	30.3%	66	66.0%	0.257	0.612
	PCU	22	64.7%	12	35.3%	34	34.0%		
Site of the center	Urban	27	79.4%	7	20.6%	34	34.0%	3.083	0.079
	Rural	41	62.1%	25	37.9%	66	66.0%		
year of experience in Family medicine	less than 5 years	36	58.1%	26	41.9%	62	62.0%	7.402	0.007*
	=5 years	32	84.2%	6	15.8%	38	38.0%		

* statistically significant P< 0.05 Fisher Exact, if cells <5

The majority of physicians in the age group of 30-35 and those with more than 5 years of experience had favorable attitude (85.7% and 84.2%) respectively. More than three quarters of female physicians, (77.5%) had favorable attitude.

Table 2 shows the relationship between personal characteristics/attitude of family physicians and their practice of infertility management at primary care.

**Table 2:** The relationship between personal characteristics/attitude of family physicians and their practice of infertility management in primary care

		Practice of infertility management						x^2^	P value
		Practice		No Practice		Total			
		N=75	%	N=25	%	N=100	%		
Age groups	24-29	47	77.0%	14	23.0%	61	61.0%	0.894 Fisher Exact	0.639
	30-35	21	75.0%	7	25.0%	28	28.0%		
	>35	7	63.6%	4	36.4%	11	11.0%		
Gender	Male	15	51.7%	14	48.3%	29	29.0%	10.730	0.001*
	Female	60	84.5%	11	15.5%	71	71.0%		
Qualification	Master degree	17	65.4%	9	34.6%	26	20.0%	1.733	0.188
	Others	58	78.4%	16	21.6%	74	11.0%		
Place of work	FPC	51	77.3%	15	22.7%	66	66.0%	0.535	0.465
	PCU	24	70.6%	10	29.4%	34	34.0%		
Site of the center	Urban	28	82.4%	6	17.6%	34	34.0%	1.485	0.223
	Rural	47	71.2%	19	28.8%	66	66.0%		
year of experience in Family medicine	less than 5 years	49	79.0%	13	60.0%	62	79.0%	1.415	0.234
	≥5 years	26	68.4%	12	40.0%	38	17.0%		
Appropriate/inappropriate place to manage infertility	Appropriate	65	84.4%	12	15.6%	77	77.0%	15.829	0.000*
	Inappropriate	10	43.5%	13	56.5%	23	23.0%		
Attitude towards processes of infertility management	Favorable attitude	55	80.9%	13	19.1%	68	68.0%	3.922	0.048*
	Unfavorable attitude	20	62.5%	12	37.5%	32	32.0%		

* statistically significant P< 0.05 Fisher Exact, if cells <5

Three quarters (75%) of the participants dealt with infertility cases. There were statistically significant difference regarding gender, perceiving PHC as an appropriate place to manage infertility and attitude towards processes of infertility management with the physicians′ practice (P < 0.05).

The majority of female physicians (84.5%), those with favorable attitude towards primary care as an appropriate place (84.4%) and those with favorable attitude towards processes of infertility management in primary care (80.9%) practiced infertility management.

Table 3 shows the frequency of the reported favorable attitude and practices of infertility management processes by the studied family physicians. In the support and proposal section: the most frequent favorable attitude was towards encouraging couples to avoid cigarettes, alcohol and drug abuse (99%) while the frequent practices were giving information about coit order (85.3%). In the investigation section: the most frequent favorable attitude and practice were for semen analysis (95% and 85.3%) respectively. In the treatment section: the most frequent favorable attitude and practice were for appropriate referral (91% and 85.3%) respectively.

**Table 3:** Frequency of reported favorable Attitude and practice of family physicians towards process s of infertility management in primary care setting

	Attitude n-100	%	Practice n= 75	%
**Supportive treatment and proposals for infertile couples**				
1. Meet the couple	10	10.0%	12	16.0%
2. Take complete history and physical examination	90	90.0%	54	72.0%
3. Begin folic acid support	88	88.0%	58	77.3%
4. Encourage couples to avoid cigarettes, alcohol and drug abuse	99	99.0%	63	84.0%
5. Resolve obesity	89	89.0%	59	78.7%
6. Prevent testicular hyperthermia	80	80.0%	38	50.7%
7. Give information about coit order	92	92.0%	64	85.3%
8. Investigate and support psychological burden	85	85.0%	58	77.3%
**Concerning request for laboratory investigations at primary care level in evaluating infertility in couples**				
9. Request Semen analyses	95	95.0%	64	85.3%
10. Midluteal S. progesterone	76	76.0%	40	53.3%
11. FSH, LH/P	89	89.0%	45	60.0%
12. Ultrasonic folliculometric	81	81.0%	25	33.3%
13. U/S diagnoses of PCOS	90	90.0%	53	70.7%
14. evaluate cases using U/S	83	83.0%	39	52.0%
15. Thyroid function	83	83.0%	36	48.0%
16. Prolactine	92	92.0%	47	62.7%
17. Adrenal hormones	81	81.0%	20	26.7%
18. Ask about previous hysterosalpingographic study	77	77.0%	38	50.7%
**Regarding treatment, education, or referral of infertile cases**				
19. Inform about hysterosalpingogram	68	68.0%	37	49.3%
20. Inform about laparoscopy	72	72.0%	21	28.0%
21. Treat sexually transmitted diseases	86	86.0%	44	58.7%
22. Referral to secondary care	91	91.0%	64	85.3%
23. Administer clomiphen citrate for ovulation induction	79	79.0%	46	61.3%
24. Administer bromocriptin for hyperprolactinemia	70	70.0%	40	53.3%
25. Treatment of hyperandrogenemia	44	44.0%	15	20.0%
26. Administer metphormine for cases with PCOS	62	62.0%	28	37.3%
27. Administer gonodotrophin for ovulation induction	50	50.0%	14	18.7%
28. Request empiric treatment for pus in semen for male	64	64.0%	23	30.7%
29. Give information about ovarian drilling	64	64.0%	20	26.7%
30. Give information about Intrauterine insemination procedure	76	76.0%	25	33.3%
31. Give information about IVF	80	80.0%	28	37.3%

IVF: In Vitro Fertilization PCOS: Polycystic Ovarian Syndrome U/S: Ultrasonography

**Figure 1:**
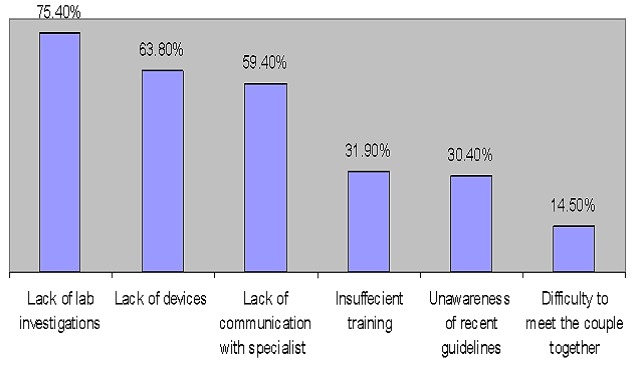
Difficulties towards infertility management at primary care

**Figure 2:**
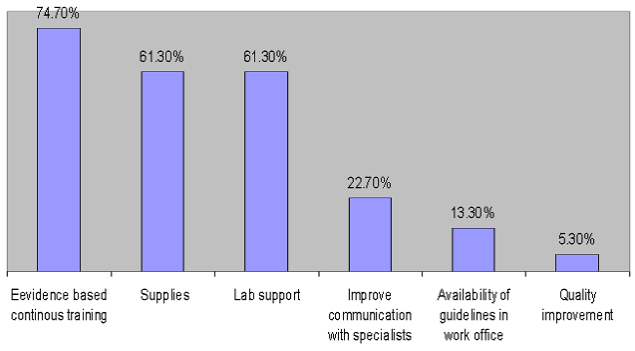
Suggestions to improve infertility management at primary care

Figure 1 shows the reasons behind difficult management of the infertile couple in primary care settings. The major reasons were, lack of laboratory investigations (75.4%), lack of devices (as U/S) 63.8% and lack of communication with a specialist after referral (59.4%). Figure 2 shows suggestions to improve management of infertility at the primary care settings. Evidence based continuous training were the most obvious suggestion by (75%) followed by providing supplies and lab support (59% and 58 %) respectively.

## Discussion

Out of 100 participants in the present study, 68 family physicians had favorable attitude towards processes of infertility management. The majority of physicians in the age group of 30-35 and those with more than 5 years of experience had favorable attitude (85.7% and 84.2%) respectively. More than three-quarters of female physicians (77.5%) had favorable attitude. This could be related to the cumulative training with age and years of experience. In addition, preference of female physicians in women's health issues could explain their favorable attitude.

More than 3 quarters of family physicians (77%) included within this study viewed the PHC as an appropriate place for infertility management. The present results were better than that found by Hassa et al [14] about attitudes to and management of fertility among primary health care physicians in Turkey and in the study by Ittner et al [8] about German family physicians' attitudes toward care of involuntarily childless patients (38.9% and 27.0%) respectively. The different results could be related to the perceived importance of infertility management by the studied sample, as it is a main topic in the curriculum of family medicine.

In the present study, (75%) of family physicians previously practiced infertility management in primary care. This was in contrast to the study by Hassa et al [14], which found that more than half of the physicians had been involved in cases of infertility (57.6%), and reported that they directly referred infertile patients to a higher-level healthcare provider. These findings could be related to the favorable attitude towards PHC as an appropriate place for the management of infertility.

The majority of female physicians practiced infertility management (84.5%). This could be explained in light of that the majority of physicians in the sample were females. Also in the present study, most of the physicians worked in rural PHC, where female patients prefer female physicians with interest of female physicians in women's health issues.

Most of the family physicians with favorable attitude regarding PHC as an appropriate place (84.4%) and those with favorable attitude (80.9%) towards the processes of infertility management at primary care practiced this service. This was in contrast to the turkey study in which only one-third of the physicians reported that primary care settings were an appropriate place for infertility management. This could be related to family physicians′ interest and knowledge regarding infertility management in the present study.

Although practice of infertility management was reported by a higher percentage of family physicians than their attitude scores, the frequency of family physicians who reported that they could perform the various processes towards infertility management at primary care was better than that of their practice.

In reviewing the frequencies of favorable attitude and practices regarding supportive treatment and proposals: except for meeting the couple, most physicians reported that they could perform a large part of these processes which ranged from (80.0%) in prevention of testicular hyperthermia to (99.0%) in smoking cessation. While physicians′ practice ranged from (50.7%) regarding behaviors towards raising sperm quality to (85.3%) regarding giving information regarding coit order. The difference showed better attitude than actual clinical practice, which could be related to the flow rates of infertility cases within PHC settings.

The present results were better than the attitude in the study by Hassa et al which ranged from (67.4%-88.6%) which were viewed as parallel to their findings about their practice during their working life. While the study by ittner et al reported an apparent underperformance by family physicians in their ability to identify psychological distress in their patients. The difference between these studies could be related to different training rather than the rate of practice.

However, in reviewing the reported attitude and practice regarding the requests for laboratory investigations at primary care level, physician reported that they could request between (76%) for progesterone and (95%) for semen analysis of the required investigations which were to some extent in parallel to practicing this issue which ranged from (26.7%) for adrenal hormones to (85.3%) for semen analysis. In the turkey study, the physicians could able to request between (43.0%) for performing U/S to (83.7%) for semen analysis). In the present study and the turkey study [14] investigation of semen analysis showed the consideration of male factor in assessment of infertility cases even if not meeting the couple which is supported in the guidelines for managing infertility.

Regarding treatment and providing information to infertile cases, physicians could perform from (44.0%) for treatment of sexually transmitted diseases to (91.0%) for appropriate referral while their practice ranged from (20.0%) for treatment of hyperandrogenemia to (85.3%) for appropriate referral. These results were not similar to that of the Turkey study [14] as physicians reported the ability to perform from (26.2%) for hormonal treatment for male factor to (92.2%) for treatment of sexually transmitted diseases. This means underperformance in the present study than in the turkey study which could be related to inadequate training in intervention processes by the studied physicians.

Two third of physicians (69%) believed that it was difficult to manage including both views of could not be managed in primary care and could be managed with difficulties which was similar to the study by Hassa et al 2005 [14].

The major reasons behind difficult management of the infertile couple at PHC were, lack of laboratory investigations in primary care, lack of devices in primary care (as U/S)3 and lack of communication with specialist after referral (75.4%, 63.8% 3& 59.4%) respectively. These results were to some extent in parallel to the study by Hassa et al [14] which found that the most frequent reason for practice difficulty was a lack of devices/equipment at primary care (55.7%). In addition, it was consistent with a study by Heyes et al [16] that reported resource constraints as a reason for similar difficulties in providing preconception care at primary care.

Training was the most obvious suggestion forwarded by family physicians who practiced infertility management in primary care by approximately (75%) followed by providing supplies and laboratory support (61.3%) for each item. This was, to some extent similar in the study by Hassa et al [14], which reported, the importance of a planned post-graduate continuing education program by (70%) and improvement in laboratory investigations by (67.2%) in the recommendations section.

**Limitations of the study:** The results of the study couldn′t be generalized to all primary care physicians as the study was conducted among family physician trainees in academic organization. There was a paucity of comparable studies.

## Conclusion

Favorable attitude scores towards processes infertility management were determined among (68%). Favorable attitude towards PHC as an appropriate place for infertility management was found among (77%). Practice of infertility management was reported by (75%). Considering the difficulties faced by family physicians and their suggestion for improvement of infertility management in the primary care settings direct the responsible authorities to improve the structure of primary care settings and provide evidence based continuous training to improve the quality of infertility management at primary care.
